# Necrotizing Soft Tissue Infections in Intensive Care

**DOI:** 10.1177/08850666211010127

**Published:** 2021-04-26

**Authors:** Alexandru Ogica, Christoph Burdelski, Holger Rohde, Stefan Kluge, Geraldine de Heer

**Affiliations:** 1 Department of Intensive Care Medicine, University Medical Centre Hamburg-Eppendorf, Hamburg, Germany; 2 Institute of Microbiology, Virology and Hygiene, University Medical Centre Hamburg-Eppendorf, Hamburg, Germany

**Keywords:** necrotizing soft tissue infection, sepsis, lactate, intensive care

## Abstract

**Background:**

Necrotizing soft tissue infections (NSTIs) are typically characterized by extensive soft tissue destruction with systemic signs of toxicity, ranging from sepsis to septic shock. Our aim was to analyze the clinical characteristics, microbiological results, laboratory data, therapies, and outcome of patients with NSTIs admitted to an intensive care unit (ICU).

**Methods:**

A monocentric observational study of patients admitted to the ICU of a university hospital between January 2009 and December 2017. The demographic characteristics, comorbidities, clinical features, microbiology and laboratory results, organ dysfunctions, therapies, and outcome were retrospectively analyzed.

**Results:**

There were 59 patients and 70% males. The mean age (± SD) was 55 ± 18; type II (monomicrobial) NSTI was present in 36 patients (61%); the most common isolated pathogen was *Streptococcus pyogenes* in 28 patients (48%). Septic shock was diagnosed in 41 patients (70%). The most common organ dysfunctions were circulatory and renal in 42 (71%) and 38 patients (64%). The mean value (± SD) of serum lactate at admission to the ICU was 4.22 ± 5.42 mmol/l, the median SOFA score and SAPS II were 7 (IQR 4 - 10) and 46 (IQR 30.5 - 53). ICU mortality rate was 25%. Both SOFA score and serum lactate demonstrated a good prognostic value regarding ICU outcome (OR 1.29, 95%CI 1.07-1.57, *P* < 0.007 and OR 1.53, 95%CI 1.19-1.98, *P* < 0.001). A cut-off value for serum lactate of 6.55 mmol/L positively predicted mortality with 67% sensitivity and 97% specificity.

**Conclusion:**

NSTIs carry a high risk of septic shock and multiple organ dysfunction syndrome and thus are still associated with high mortality. In our study, the value of serum lactate at admission to the ICU correlated well with mortality. This easy-to-measure parameter could play a role in the decision-making process regarding prognosis and continuation of care.

## Introduction

Necrotizing soft tissue infections (NSTIs) include necrotizing forms of fasciitis, myositis, and cellulitis and are divided into 2 groups, referring to microbiological findings: type I polymicrobial and type II monomicrobial.

Worldwide incidence varies from 0.3 to 5 cases in Europe^1,2^ and up to 15.5 cases per 100,000 inhabitants in Asia.^3^ The reported mortality rates range from 20% to 32%,^1,4^ with an average of 20% within the past 20 years.^5^

Necrotizing soft tissue infections are medical emergencies characterized by extensive soft tissue destruction. The clinical picture ranges from mild sepsis to septic shock. Various degrees of organ dysfunction are common during this period.^6^ Early surgical exploration and extensive antibiotic therapy form the cornerstones of therapy. The first 24 hours after surgery in the intensive care unit (ICU) are critical, as the postoperative cytokine storm results in a global nonspecific inflammatory reaction characterized by vasodilation, microcirculatory disturbance, and capillary leak.

Previous studies demonstrated conflicting results concerning predictors for mortality in these patients. Furthermore, detailed information regarding the course of the disease in the ICU is lacking.

In the present study, we extensively describe the intensive care course of these patients and search for possible predictors for mortality through statistical analysis.

## Material and Methods

### Study Design

We retrospectively described a cohort of critically ill patients treated for an NSTI at one of the ICUs of our university hospital between January 2009 and December 2017.

### Setting

The University Medical Centre Hamburg-Eppendorf is a tertiary-level metropolitan medical center, containing 1,738 hospital beds and reaching a volume of more than 100,000 in-patients per year. The Department of Intensive Care Medicine includes 12 ICUs, with a total capacity of 140 ICU beds. It serves the maximum level of treatment to medical and surgical patients.

### Participants and Data Collection

We included all patients aged 18 or older, who were admitted to the ICU for NSTI. The follow-up period ended with the discharge from the hospital. Patients with non-necrotizing soft tissue infections (e.g. abscesses or phlegmons) were excluded. Data were provided by the electronic patient data management systems [Soarian, Siemens, Erlangen for non-ICU data and Integrated Care Manager (ICM), Dräger Medical, Lübeck, Germany for ICU data]. Disease characteristics (comorbidities, risk factors, localization, imaging, surgical interventions, and microbiologic characteristics) and ICU data (laboratory values at admission to the ICU, organ dysfunctions, shock parameters, and medical therapy) were extracted. The Laboratory Risk Indicator for Necrotising Fasciitis score (LRINEC score), a disease-specific score proposed by Wong et al^7^ to distinguish NSTIs from other soft tissue infections, was computed retrospectively. The baseline laboratory values recorded at admission to the ICU were used to quantify the degree of organ dysfunction and in calculating the sepsis-related organ failure assessment (SOFA) score at admission. We employed the most recent definitions for sepsis and septic shock proposed by the 3rd Surviving Sepsis Campaign.^8,9^ Circulatory dysfunction was defined by the use of vasopressors. Acute kidney injury was classified using the KDIGO Guidelines (“Kidney Disease—Improving Global Outcomes”).^10^ Hematological, liver, neurological und pulmonary dysfunction were defined according to SOFA score definitions. The severity of illness was assessed using the Simplified Acute Physiology Score II (SAPS II) at 24 hours after admission to the ICU. Serum lactate, vasopressor use, and fluid balance were documented 24 hours after admission to the ICU, as well.

### Statistics

Patients’ data were collected using Excel (Microsoft Software, Version 2013) and presented either as absolute numbers with percentages or as a median, range, interquartile range (IQR), or mean (± standard deviation), as appropriate. Student’s *t*-test was used for continuous variables, while Pearson’s chi-square or Fisher’s exact test was used for categorical variables, as appropriate. Parameters were considered significant in the univariate analysis if the 2-tailed *P*-value was less than 0.05. The endpoint was ICU mortality. For the clinically relevant variables, which demonstrated significant correlation to the selected endpoint, we computed receiver operating characteristic (ROC) curves. Statistical analysis was conducted using IBM SPSS Statistics Version 25.0.

## Results

During the study period, 59 patients were treated for an NSTI in the ICUs of our hospital.

### Demographic Data

The mean age (± SD, range) was 55 ± 18 years (19 to 89), and the proportion of male patients was 70%. The most frequent comorbidity was arterial hypertension, found in 21 patients (36%). Moreover, various types of autoimmune diseases were present in 12 patients (20%). Table 1 provides a detailed list of the patients’ demographic characteristics and comorbidities.

**Table 1.** Demographic Characteristics and Comorbidities of the Patients With NSTI, Classified by Outcome.

Variable	Patients (N = 59)	ICU survivors (N = 44)	ICU non-survivors (N = 15)	*P* value
Male gender (N, %)	41 (70)	30 (68)	11 (73)	NS
Age, y.o. (mean ± SD)	55 ± 18	54 ± 18	58 ± 18	NS
Weight, kg (mean ± SD)	85 ± 24	83 ± 21	90 ± 31	NS
BMI, kg/m^ 2 ^ (mean ± SD)	27 ± 6	27 ± 6	28 ± 6	NS
Comorbidities (N, %) Arterial hypertension Immunosuppressive therapy Autoimmune disease History of cancer Diabetes mellitus Peripheral vasculopathy Coronary artery disease Hypercholesterolemia Liver disease COPD Alcoholism	21 (36) 16 (27) 12 (20) 12 (20) 11 (19) 8 (14) 8 (14) 6 (10) 5 (8) 5 (8) 5 (8)	14 (32) 11 (25) 6 (14) 10 (23) 7 (16) 4 (9) 6 (14) 4 (9) 0 2 (5) 4 (9)	7 (47) 5 (33) 6 (40) 2 (13) 4 (27) 4 (27) 2 (13) 2 (13) 5 (33) 3 (20) 1 (7)	NS NS 0.02 NS NS NS NS NS NS NS

Abbreviations: BMI, body mass index; COPD, chronic obstructive pulmonary disease; NS, not significant.

### Admission Data

All patients arrived through the emergency department, 19 (32%) as an interhospital transfer. Chronic wounds were observed in 12 patients (20%), while 11 (20%) developed an NSTI postoperatively and 10 (17%) after a skin breach through injections or minor trauma. The classical triad of pain, swelling, and erythema was present in only 7 patients (12%). The major localization of the NSTI was the lower extremity in 41 patients (70%).

Diagnostic imaging studies were performed in 38 patients (64%). This was associated with a higher, but statistically not significant, percentage of patients receiving surgical exploration within the first 6 hours (71% versus 52%, *P*-value 0.08).

The median value (IQR) of the LRINEC score was 7 points (5-8), 6 points (6-8) for survivors and 7 points (5-8) for non-survivors. The proposed cut-off of 6 points was present in 42 patients (72%).

### Surgical Therapy

All patients received at least one surgical debridement within the first 24 hours after establishing the diagnosis, 38 of whom (67%) within 6 hours after hospital admission. A subsequent debridement was necessary in 22 patients (44%). This was dictated by clinical worsening in 13 patients (59%) and planned second look surgery for the others. The median (IQR) number of surgical interventions including plastic surgery until discharge was 4 (2-6). Limb amputation and disarticulation was necessary in 8 patients (17%). The surgical intervention within the first 6 hours after admission did not influence the rate of amputation (*P*-value 0.84).

### Antibiotic Therapy and Microbiological Data

There were 17 patients (29%) with type I and 36 patients (61%) with type II NSTI. In 6 patients (10%), no pathogen was isolated. The antibiotic regimen received at presentation or before admission was empirically modified in 35 patients (59%) within the first 24 hours. The antibiotic regimen has been changed for several reasons, often because the diagnosis of NSTI was not clear initially, or addition of a further antibiotic (vancomycin, clindamycin etc.) has been performed.

Complete data concerning these general aspects and therapy are provided in Table 2.

**Table 2.** Medical Data of the Patients Classified by Outcome.

Variable (N, %)	Patients (N = 59)	ICU survivors (N = 44)	ICU non-survivors (N = 15)	*P* value
Risk factors				
Chronic wounds	13 (22)	8 (18)	5 (33)	NS
Skin breach	10 (17)	9 (21)	1 (7)	NS
Trauma	4 (7)	3 (7)	1 (7)	NS
Postoperative	12 (20)	10 (23)	2 (13)	NS
Signs and symptoms at presentation				
Fever	9 (15)	9 (21)	0	NS
Pain	31 (53)	25 (57)	6 (40)	NS
Swelling	33 (56)	25 (57)	8 (53)	NS
Erythema	25 (42)	20 (46)	5 (33)	NS
Gastrointestinal symptoms	10 (17)	7 (16)	3 (20)	NS
Bullae and skin necrosis	9 (15)	5 (11)	4 (27)	NS
NSTI Iocalisation				
Lower extremity	41 (70)	31 (71)	10 (67)	NS
Upper extremity	6 (10)	5 (11)	1 (7)	NS
Head and neck	7 (12)	4 (9)	3 (20)	NS
Thoracoabdominal	7 (12)	5 (11)	2 (13)	NS
Imaging studies				
Computer tomography	22 (40)	14 (34)	8 (57)	NS
Magnetic resonance imaging	16 (29)	14 (34)	2 (14)	NS
Surgical therapy				
Intervention within 6 hours after admission	38 (66)	28 (64)	10 (71)	NS
Second exploration within the next 24 hours	22 (42)	14 (33)	8 (80)	0.007
Limb amputation with disarticulation	8 (17)	5 (11)	3 (20)	NS
Less than 4 surgical interventions	21 (36)	11 (25)	10 (67)	0.004
Antibiotic therapy				
Initial combination therapy^a^	32 (54)	21 (48)	11 (73)	NS
Initial clindamycin	36 (61)	26 (59)	10 (67)	NS
Positive blood cultures	8 (15)	5 (13)	3 (21)	NS
Microorganism				
* Streptococcus pyogenes*	28 (48)	23 (52)	5 (33)	NS
* Streptococcus dysgalactiae*	6 (10)	4 (9)	2 (13)	NS
* Staphylococcus aureus*	11 (19)	7 (16)	4 (27)	NS
* E. coli*	9 (15)	4 (9)	5 (33)	0.02
* Acinetobacter spp.*	3 (5)	3 (7)	0	NS
* Pseudomonas aeruginosa*	3 (5)	3 (7)	0	NS
* Enterobacter spp.*	3 (5)	0	3 (20)	0.01

Abbreviation: NS, not significant.

^a^Combination therapy - carbapenem/acyl-aminopenicillin plus a beta-lactamase inhibitor and a glycopeptide or linezolid.

### ICU Data

The median SOFA score was 7 (IQR 2-10) for survivors and 9 (IQR 8-12) for non-survivors, while the median SAPS II after 24 hours was 42 (IQR 27-51) for survivors and 53.5 (IQR 50-59) for non-survivors.

Septic shock was present in 41 patients (70%). The most frequent organ dysfunctions were circulatory in 42 (71%) and renal in 38 patients (59%), respectively. The presence of more than 4 organ dysfunctions was significantly linked to a higher mortality (*P*-value 0.04). The details are presented in Table 3.

**Table 3.** Distribution of Septic Shock and Organ Dysfunctions at Admission, Classified by Outcome.

Variable (N, %)	Patients (N = 59)	ICU survivors (N = 44)	ICU non-survivors (N = 15)	*P* value
Septic shock	41 (69)	26 (59)	15 (100)	0.003
Circulatory dysfunction	42 (71)	27 (61)	15 (100)	0.004
Renal dysfunction	38 (64)	26 (59)	12 (80)	0.002
Haematological dysfunction	29 (49)	21 (48)	8 (53)	NS
Liver dysfunction	16 (27)	6 (14)	10 (67)	< 0.001
Neurological dysfunction	8 (14)	5 (11)	3 (20)	NS
Pulmonary dysfunction	7 (12)	4 (9)	3 (20)	NS

Abbreviation: NS, not significant.

The relationship between laboratory values and mortality revealed an incongruous pattern. Serum lactate, C-reactive protein (CRP), and prothrombin time (PT) significantly differed between the 2 groups, as presented in Table 4. The CRP was considerably lower in the group of patients with 4 or more organ dysfunctions (11 patients—19%, *P*-value 0.05), however, this did not reach statistical significance.

**Table 4.** Selected Laboratory Values at Admission, Classified by Outcome.

Variable (mean ± SD)	Patients (N = 59)	ICU survivors (N = 44)	ICU non-survivors (N = 15)	*P* value
WBC count, x 10^ 3 ^/µL	15 ± 11	13 ± 7	18 ± 16	NS
Thrombocyte count, x 10^ 3 ^/µL	182 ± 156	186 ± 171	170 ± 103	NS
Lactate, mmol/L	4.2 ± 5.4	2.1 ± 1.7	10.3 ± 7.6	< 0.001
CRP, mg/dl	209 ± 100	226 ± 104	157 ± 69	0.02
PCT, ng/dl	24 ± 28	22 ± 26	31 ± 34	NS
CK, IU/L	3,284 ± 10,781	6,126 ± 12,926	2,132 ± 9,745	NS
Prothrombin time, sec	70 ± 26	74.41 ± 25.84	55.66 ± 21.96	0.015
Creatinine, mg/dl	2.29 ± 1.68	2.17 ± 1.64	2.64 ± 1.82	NS

Abbreviations: WBC, white blood cells; CRP, C-reactive protein; PCT, procalcitonin; CK, creatine kinase; NS, not significant.

Norepinephrine was the most frequently administered vasopressor, received by 40 patients (68%). An increase in the requirement for catecholamine support within the first 24 hours after admission to the ICU was observed in 19 patients (32%). Nine patients (15%) required additional inotropic therapy.

During the first 24 hours, 37 patients (63%) were invasively ventilated. Mechanical ventilation for more than 24 hours was necessary for 30 patients (51%).

An acute kidney insufficiency (AKI) Stage I was observed in 16 patients (27%), AKI Stage II in 13 patients (22%), and AKI Stage III in 9 patients (15%). Continuous renal replacement therapy was necessary for 26 patients (44%). Overall, there was a median positive fluid balance of approximately 5 liters over the first 24 hours. Non-survivors received in median 3.2 L more than survivors did (7.8 L versus 4.5 L). A higher positive fluid balance was directly proportional to mortality (*P*-value 0.003).

Corticosteroid therapy was administered to 24 patients (41%), and 6 patients (10%) received intravenous immunoglobulins.

A detailed presentation of the therapeutic interventions within the first 24 hours is provided in Table 5.

**Table 5.** Medical Therapy in ICU During the First 24 h, Classified by Outcome.

Variable (N, %)	Patients (N = 59)	ICU survivors (N = 44)	ICU non-survivors (N = 15)	*P value*
Vasopressor therapy	40 (68)	25 (61)	15 (100)	0.002
Inotropic therapy	9 (15)	5 (11)	4 (27)	NS
Invasive mechanical ventilation	37 (63)	23 (52)	14 (93)	0.005
Renal replacement therapy	20 (34)	9 (20)	11 (73)	< 0.001
Packed red blood cells	18 (31)	11 (25)	7 (47)	NS
Fresh frozen plasma	21 (36)	13 (30)	8 (53)	NS
Platelet concentrates	7 (12)	4 (9)	3 (20%)	NS
Corticosteroid therapy	24 (41)	12 (27)	12 (80)	< 0.001
Intravenous immunoglobulin therapy	6 (10)	5 (11)	1 (7)	NS
Enteral/parenteral nutrition	22 (37)	15 (34)	7 (47)	NS

Abbreviation: NS, not significant.

### Clinical Course

The median (IQR) length of stay in the ICU was 5 days (IQR 3-15). Fifteen patients died during ICU stay, resulting in a mortality rate of 25%. Death occurred within the first 48 hours in 8 patients (54%). In the survivor group, the median length of stay at the hospital was 35 days IQR (10-51). Three patients died in the normal ward, accounting for an in-hospital mortality rate of 30%. Table 6 presents the odds for ICU mortality (OR, 95%CI) for the significant variables after the univariate analysis.

**Table 6.** Odds Ratio for Death After the Univariate Analysis of Statistically Significant Data of the Cohort.

Variable	OR (95%CI)
Autoimmune disease Second surgical intervention within the first 24 hours E. coli isolates Presence of septic shock SOFA Score SAPS II Score Lactate at admission Failure of lactate clearance at 24 hours Circulatory dysfunction at admission Rise in norepinephrine dosage at 24 hours Liver dysfunction Corticosteroid therapy within 24 hours Renal replacement therapy ≥ 4 Organ dysfunctions	4.22 (1.1-16.2) 7.71 (1.44-41.33) 5.00 (1.13-22.10) 1.69 (1.32-2.16) 1.29 (1.07-1.57) 1.07 (1.01-1.13) 1.53 (1.19-1.98) 8.75 (2.06-37.08) 1.55 (1.24-1.94) 12.37 (3.12-49.05) 12.67 (3.19-50.15) 10.67 (2.56-44.50) 10.69 (2.74-41.61) 5.13 (1.74-15.11)

Serum lactate at admission to the ICU was a good independent factor for predicting ICU mortality. A value equal to or greater than 2 mmol/L increased the risk of mortality tenfold (95%CI 2.07-51.52, *P*-value 0.001) and greater than 4 mmol/L twentyfold (95%CI 4.52-88.4, *P*-value < 0.001). Interhospital transfer or time to surgery did not influence the value of serum lactate.

**Figure 1.**
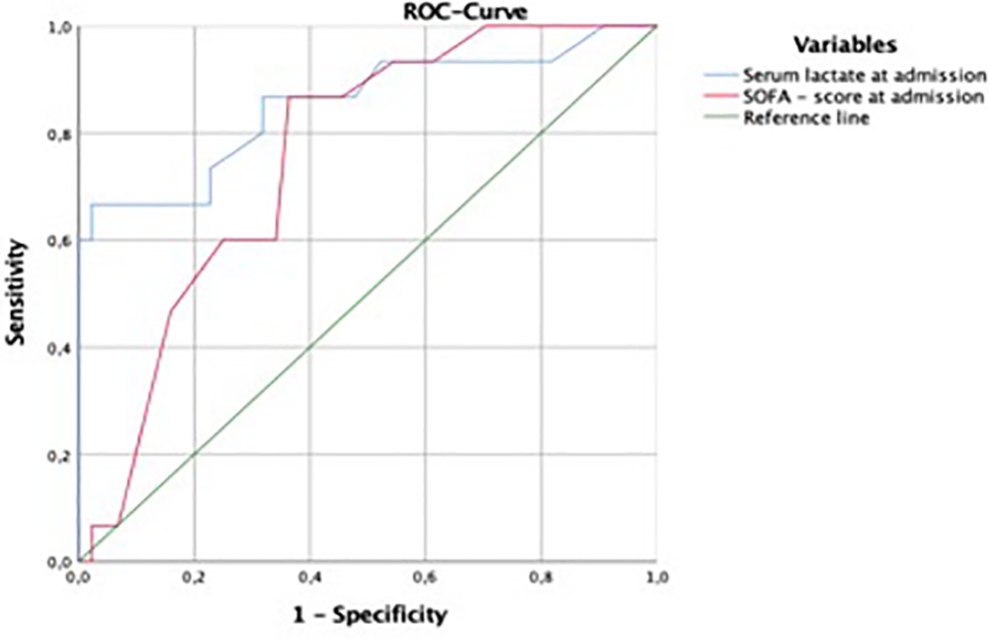
Area under the curve for serum lactate and SOFA score at admission against mortality in patients with NSTI.

Serum lactate achieved an area under the curve (AUC) slightly larger than the SOFA score (0.85 versus 0.75, *P*-value < 0.001 versus 0.003) for predicting mortality. A cut-off value for serum lactate was 6.55 mmol/L, demonstrating 67% sensitivity and 97% specificity in positively predicting ICU mortality (see Figure 1).

## Discussion

The present study analyzed the characteristics and outcomes of patients with an NSTI. Furthermore, the potential existence of risk factors linked to mortality was investigated. We identified serum lactate at admission as a robust parameter for predicting mortality. To our knowledge, our study is the first European study to link the value of serum lactate at admission to the ICU with mortality. In addition, we conducted a detailed analysis of intensive care therapy regarding, primarily, the first 24 hours after admission.

NSTIs are rare medical entities, especially in the Western world, so most monocentric studies in Europe and the US contain small numbers of patients over long periods.^4,11–13^ Multiple parameters predicting a negative outcome have been proposed.

Our cohort included 59 patients over a period of 9 years. In this time period 73,418 patients have been treated at our department of intensive care, resulting in a mean admission rate of 0.08%. The ICU and in-hospital mortality rates were 25% and 30%, respectively—comparable with those from previous studies conducted during the past 2 decades^1,4,7,11,13–18^ and slightly lower compared with earlier studies. A recently published metanalysis using 109 studies presented a mean mortality of 21%.^5^ Our slightly higher mortality could be explained in that, being a university hospital, our unit receives the most critically ill patients from north Germany. One-third of the patients in our cohort were admitted by interhospital transfer.

Eight patients exhibited a fulminant course and died within the first 48 hours after admission to the ICU due to overwhelming septic shock with multiple organ dysfunction syndrome. Van Stigt et al reported a similar percentage,^13^ again confirming the severity of this disease.

Our study contained the largest proportion of patients with autoimmune diseases to date. This was a significant risk factor for mortality in the univariate analysis, increasing the odds of dying by a factor of 4. We suppose that alterations of the immune system caused by the disease itself or by its therapy might favor NSTIs in such patients. The literature concerning this association is scarce and exists primarily in the form of case reports.^19^

The statistical analysis revealed no statistically relevant association between the remaining demographic data, risk factors, clinical presentation or localization of the NSTI, and mortality compared with other studies.^4,20,21^ Different settings and cohorts could be responsible for such discrepancies.

The LRINEC score helps to differentiate between necrotizing and non-necrotizing forms of soft tissue infections^7^ but has been subject to criticism over the years.^22^ This score is not routinely used in our hospital, so it was computed retrospectively for descriptive purposes. Its addition to a carefully recorded patient’s history with an unclear clinical presentation could be valuable in increasing diagnostic chances.^23^

Surgical exploration was performed within the first 6 hours after hospital presentation in 38 patients (64%). Although our study did not reveal any statistically relevant reduction in mortality in these patients, it is our strong belief, in line with a recent meta-analysis,^5^ that the surgical debridement is a treatment of highest priority in NSTI and should be performed without any delays. Some patients had already undergone a surgical intervention in the first hospital before being transferred, others were preoperatively admitted to the ICU for hemodynamic improvement, and others still had undergone a small debridement in the emergency department, which was not mentioned as a true surgical intervention in the documentation. Based on the small number of patients in each situation, it is not possible to draw a definite conclusion regarding such practices.

Regarding amputations in NSTIs, the recently published study by Chang et al had an amputation rate of 6% and classified amputations as early and late according to a cut-off time of 3 days.^24^ This procedure was performed within 3 days in all patients in our study. There was no significant association between the timing of the first surgical exploration and the rate of amputation, as was demonstrated in a recent meta-analysis.^5^

The presence of *Escherichia coli* or *Enterobacter spp*. in the wound isolates was associated with higher odds of increased mortality in the univariate analysis. To our best knowledge, studies that found significant correlations between different microorganisms and outcome are scarce. In a large Taiwanese study, infection with *Aeromonas spp.* was an independent factor for increased mortality.^21^ In a recently published study, infection with Streptococcus pyogenes of Group A was associated with a significantly lower mortality.^25^ Both *E.coli* and *Streptococcus pyogenes* were the most frequent microorganisms in a large study from Thailand, but there was no correlation to the outcome.^20^ NSTIs with *Enterobacter spp* appear sporadically in the form of case reports.^26,27^ The 2 bacteria typically appeared in type I (polymicrobial) NSTI, seen mostly postoperatively in patients with multiple comorbidities. Small numbers make it difficult to draw definite statements; however, it stresses once again the necessity for combination antibiotic therapy from the moment of NSTI suspicion.

Both the SOFA score at admission to the ICU and the SAPS II score at 24 hours after admission correlated well with mortality. Circulatory and renal dysfunction were the most common organ dysfunctions during the first 24 hours. We identified a statistically significant association between initial circulatory and liver dysfunction and mortality in the univariate analysis. The current literature is not consistent on this matter: a study by Krieg et al identified AKI as an independent prognostic parameter for mortality,^4^ while Khamnuan et al revealed no correlation between organ dysfunction and mortality.^3^ Demographic differences could explain these discrepancies; for example, the number of patients with diabetes mellitus in the first study was significantly higher compared to ours (42% versus 19%). The second study had a surprisingly low mortality rate (9.8%), and only 16% of patients had some form of organ dysfunction.

We interpreted the lower C-reactive protein levels in the subgroup of non-survivors more as a sign of deterioration of the liver function and synthesis than as an overall exhaustion of the immune response.

Signs of refractory septic shock with multiple organ dysfunctions as ongoing circulatory dysfunction, a necessity for renal replacement therapy or corticosteroid therapy and a positive fluid balance after the first 24 hours, were significantly associated with higher mortality, all of which were markers of disease severity. Septic shock is still associated with high mortality regardless of the cause, with a systematic review and meta-analysis demonstrating mortality at around 38% across Europe and North America.^28^

Similar to the results of another study,^29^ the present study could not establish a benefit of using intravenous immunoglobulin therapy. Since only a small number of patients received immunoglobulins, we cannot draw any definitive conclusion. At the moment, there is no recommendation concerning their utilization for the treatment of NSTIs.^6^

The major finding of the present study was the identification of serum lactate at admission to the ICU as being a good predictor for mortality. This observation is consistent with recent studies linking severe hyperlactatemia in general critically ill patients to mortality.^30,31^ The failure of serum lactate clearance after 24 hours was a negative predicting factor for survival in the univariate analysis, as well. Computing the ROC curves for serum lactate and the SOFA score, we observed better performance of the former in predicting mortality. Our results are similar to a recently published study by Chang et al.^32^ In the present study, the serum lactate achieved a slightly larger AUC than the SOFA score.

Murphy et al demonstrated in a small study that serum lactate at presentation could serve as a marker to differentiate NSTIs from other non-necrotizing soft tissue infections, with lactate serving as a potential marker for tissue necrosis.^33^

Linking hyperlactatemia to the prediction of mortality is an important finding in our study. Serum lactate is a simple parameter that can be easily measured by most point-of-care machines in the emergency department or in the ICU. A higher value could be helpful in the decision-making process, regarding further interventions or even discontinuation of care.

Our study has several limitations. It was a monocentric retrospective study with a limited number of patients over 9 years. The small number of patients and therefore small number of unfortunate events limited the use of advanced statistic studies, such as multivariate regression analysis. Concerning serum lactate, we used the first measured value upon admission to the ICU. As some of the patients were initially stable or were classified as having only an isolated dermatological problem, serum lactate was not routinely measured in the emergency department. We are aware that the admission value for serum lactate could have been influenced by a variety of factors. Nevertheless, and in line with recent studies, serum lactate continues to prove itself as a useful cellular marker, with higher dynamic values typically suggesting a greater threat to the patient’s vital status. Future prospective well-conducted studies should further explore the relationship between serum lactate at admission to the emergency department and mortality before any therapeutic actions are taken.

## Conclusion

Necrotizing soft tissue infections are major surgical and intensive care emergencies. They progress rapidly to septic shock with multiple organ dysfunction syndrome and are still associated with high mortality. The fulminant course of the disease was evident in our study, with 8 patients dying within the first 48 hours after admission to the ICU. We identified serum lactate at admission as a good prognostic marker for ICU mortality. Its raised value should alert the clinician about the severity of the disease and the potential for higher mortality. Moreover, it can be used as a reference value in the decision-making process regarding prognosis and continuation of care.

## Abbreviations

95%CI, 95% confidence interval; AKI, acute kidney injury; AUC, area under the curve; BMI, body mass index; CK, creatine kinase; COPD, chronic obstructive pulmonary disease; CRP, C-reactive protein; CT, computer tomography; E. coli, Escherichia coli; ICM, integrated care manager; ICU, intensive care unit; IQR, interquartile ratio; KDIGO, Kidney Disease—Improving Global Outcomes; LRINEC, Laboratory Risk Indicator for Necrotising Fasciitis score; MRI, magnetic resonance imaging; N, number of patients; NSTI, necrotising soft tissue infection; OR, odds ratio; PCT, procalcitonin; PT, prothrombin time; ROC, receiver operating characteristic; SAPS II, Simplified Acute Physiology score II; SD, standard deviation; SOFA, sepsis-related organ failure assessment score; SPSS, statistical package for the social sciences; WBC, white blood cells.
